# A Case of Seronegative Pediatric Neurobrucellosis Presenting With Ataxia

**DOI:** 10.7759/cureus.12540

**Published:** 2021-01-06

**Authors:** Salman S Qasim, Salim Baharoon, Laila Layqah

**Affiliations:** 1 Medicine, King Saud bin Abdulaziz University for Health Sciences, Riyadh, SAU; 2 Internal Medicine, King Abdulaziz Medical City, Riyadh, SAU; 3 Pharmacy, King Abdullah International Medical Research Center, Riyadh, SAU

**Keywords:** brucellosis, neurobrucellosis, pediatric, ataxia

## Abstract

Neurobrucellosis is an uncommon and dangerous complication of brucellosis. Meningitis is the most common presentation of neurobrucellosis, but it may present in a wide range of clinical manifestations such as myelitis, brain abscess, radiculopathy, and cranial nerve involvement. It tends to present insidiously with symptoms appearing gradually. Acute presentation of neurobrucellosis is very uncommon. Here, we report a case of a female child who presented with an acute onset of ataxia and slurred speech with positive cerebrospinal fluid (CSF) and neuroimaging findings for neurobrucellosis. In endemic countries such as Saudi Arabia, neurobrucellosis should be considered as a differential diagnosis for patients presenting with unexplained neurological symptoms.

## Introduction

Brucellosis, a multisystem disease, is considered the most globally prevalent zoonotic infection [1]. It has been reported in many parts of the world, including the Mediterranean Basin and countries in the Middle East, such as Saudi Arabia, in which brucellosis is endemic [2,3]. Brucellosis is transmitted to humans through contact with contaminated animal tissues, such as blood and urine, or through the consumption of unpasteurized milk [4]. Brucellosis has a wide range of clinical presentations including fever, muscle and joint pain, headache, loss of appetite, rashes, and diarrhea [5]. Patients with complicated diseases may present with neurobrucellosis, an uncommon and serious complication affecting 3%-5% of cases globally [6]. The diagnostic criteria of neurobrucellosis and the subsequent treatment regimen are controversial. However, neurological symptoms with positive cerebrospinal fluid (CSF) and imaging findings increase the likelihood of establishing a correct diagnosis of neurobrucellosis after excluding all other potential causes of the disease. Here, we report a case of a female child who presented with ataxia and slurred speech with positive CSF and neuroimaging findings for neurobrucellosis.

## Case presentation

A nine-year-old girl presented to the emergency department (ED) with ataxia and slurred speech. Shortly after she arrived at the hospital, she had two episodes of generalized tonic-clonic seizures, each lasting for one minute, that were successfully treated with lorazepam and phenytoin. The patient’s family indicated a positive history of raw camel milk consumption for two years with a history of brucellosis when the patient was younger. The patient was recently discharged and treated for pharyngitis that was associated with fever and vomiting. Cardiovascular, respiratory, and gastrointestinal examinations were all normal. The neurological examination indicated right-sided weakness with intact cranial nerves. The patient was transferred to the pediatric intensive care unit (PICU) due to a reduced level of consciousness and was given intravenous (IV) ceftriaxone, vancomycin, and acyclovir empirically for suspected central nervous system (CNS) infection. During her admission, her gait had improved, and she was able to ambulate without assistance. However, on the third day of admission, she had an episode of vomiting and became irritable, complaining of neck pain, headaches, and photophobia. Subsequent physical examination revealed positive Brudzinski’s and Kernig’s signs.

**Figure 1:**
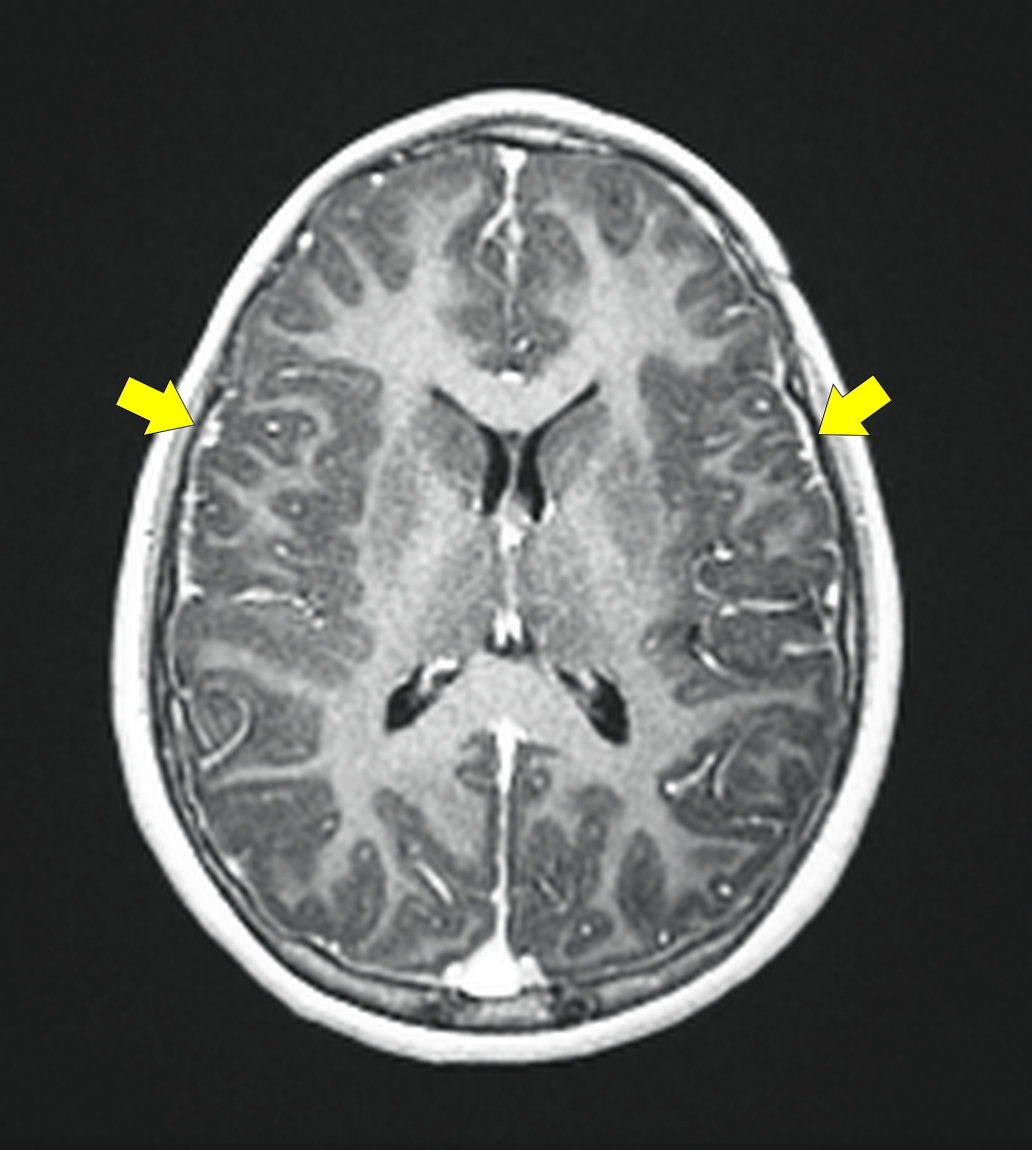
Axial post-contrast T1-weighted brain MRI showing diffuse leptomeningeal enhancement (yellow arrows) MRI: Magnetic resonance imaging.

Early laboratory tests were as follows: Full blood count showed a white blood cell (WBC) count of 5.68 x 10^9^/L, hemoglobin concentration of 117 g/L, and platelet count of 304 x 10^9^/L. Her erythrocyte sedimentation rate (ESR) was 44 mm/hr. Renal and liver function tests were normal. Blood and urine cultures were all negative. An electroencephalogram (EEG) was obtained and showed abnormal slow waves, indicating encephalopathy. A brain magnetic resonance imaging (MRI) indicated diffuse leptomeningeal enhancement with no intracranial complications (Figure 1). A lumbar puncture (LP) was performed on the same day of admission but was traumatic and inconclusive. Therefore, a repeated LP was performed and showed clear CSF with a WBC count of 17 cell/mm^3^ (lymphocytes 94%), glucose 5 mg/dL, and protein 1.48 mg/dL, suggestive of bacterial meningitis. CSF tuberculosis (TB) polymerase chain reaction (PCR), acid-fast bacilli (AFB), and CSF cultures were all negative. CSF viral PCR test was negative; thus, acyclovir was discontinued. CSF *Brucella melitensis* serum agglutination test (SAT) titer was positive at 1:10, but the serological SAT titer for *Brucella *was negative. A sample of CSF was sent to a specialized laboratory for the detection of *Brucella *species using a PCR test and came back negative. However, the sample used to perform this test was obtained while the patient was on ceftriaxone for a few days, which may have led to an inaccurate result. The diagnosis of neurobrucellosis was made due to positive CSF SAT titer for *B. melitensis* and positive neurological symptoms with neuroimaging findings.

The patient was transferred to the pediatric general ward and was started on triple therapy for the treatment of neurobrucellosis. Administration of IV ceftriaxone was continued with the addition of doxycycline and rifampicin for two weeks. During her admission, she showed significant clinical improvement with improved gait and speech and no permanent neurologic sequelae. The patient was discharged with oral antibiotics for two months and was followed up in the outpatient clinic after two weeks and then two months later.

## Discussion

The involvement of the CNS in brucellosis is an uncommon but serious complication of the disease. Neurobrucellosis has a wide range of clinical presentations. It may mimic various neurological pathologies such as myelitis, brain abscess, radiculopathy, cranial nerve involvement, subarachnoid hemorrhage, and meningitis, the latter being the most common [7]. In endemic countries, neurobrucellosis should be on top of the physician’s differential diagnoses for patients presenting with unexplained neurological manifestations. Early detection and treatment of neurobrucellosis prompt complete remission and the prevention of permanent neurological sequelae. Neurobrucellosis tends to present in an insidious course with its symptoms presenting gradually [6]. Acute presentations of neurobrucellosis are very unusual. Our patient presented with ataxia that developed the day before her arrival to the ED, with no history of previous neurological symptoms. Initially, her gait improved with empirical treatment for suspected CNS infection; but soon after, her condition suddenly deteriorated with physical examination showing positive signs of meningitis. The diagnosis of neurobrucellosis can be very challenging as there is no consensus on its diagnostic criteria. In our patient, blood culture and serological SAT titer for *Brucella *were negative. CSF analysis showed a picture of bacterial meningitis with negative culture for *Brucella *but a positive SAT titer. An EEG and a brain MRI showed abnormal slow waves and diffuse leptomeningeal enhancement, respectively. The diagnosis of our patient was made based on positive neurological symptoms, neuroimaging findings, and a positive CSF SAT titer for *B. melitensis*. Triple therapy with IV ceftriaxone, doxycycline, and rifampicin showed an excellent response and clinical improvement. The presented case was part of a retrospective cohort study that aimed to describe the clinical presentations and treatment strategies of brucellosis among Saudi children [8].

## Conclusions

Neurobrucellosis should be considered as a differential diagnosis for unexplained neurological symptoms, particularly in endemic countries such as Saudi Arabia. Taking a proper history of raw milk consumption, with a high index of suspicion, is essential in identifying the disease in a timely manner. Negative serological tests for brucellosis should not exclude the diagnosis of the disease as a CSF SAT titer for *Brucella *may be the only positive laboratory finding specific for the disease, as in our case. Prompt diagnosis and treatment will significantly reduce the chance of developing permanent neurologic sequelae in such cases.
